# Aggressive presentation of a thymosarcoma with invasion of right heart chambers: a multimodal imaging approach

**DOI:** 10.1093/ehjcr/ytae699

**Published:** 2025-01-17

**Authors:** Víctor Manuel López Espinosa, Antonio Esteban Arriaga Jiménez

**Affiliations:** Department of Cardiology, Hospital Virgen de las Nieves, Avenida de las Fuerzas Armadas, 2, 18014 Granada, Spain; Department of Cardiology, Hospital Virgen de las Nieves, Avenida de las Fuerzas Armadas, 2, 18014 Granada, Spain

A 66-year-old hypertensive and dyslipidaemic woman presented with asthenia, epigastric abdominal pain, loss of appetite, and a 5 kg weight loss over 3 months, along with exertional dyspnoea and nocturnal facial plethora. An abdominal ultrasound was initially performed due to predominantly digestive symptoms and a slight elevation of transaminases, which suggested an abdominal origin. However, the ultrasound incidentally revealed a large hyperechogenic mass in the right atrium. This finding shifted the diagnostic focus toward evaluating the cardiac mass as the cause of a systemic condition, with the initial differential diagnosis, based on echocardiographic characteristics, being between atrial myxoma and thrombus. Further multimodal imaging techniques (*Figure 1;*  Supplementary material online, *Video S1*) confirmed a high-grade thymic tumour, ultimately diagnosed as thymosarcoma infiltrating the superior cava vein (SCV) and right cardiac chambers.

**Figure 1:**
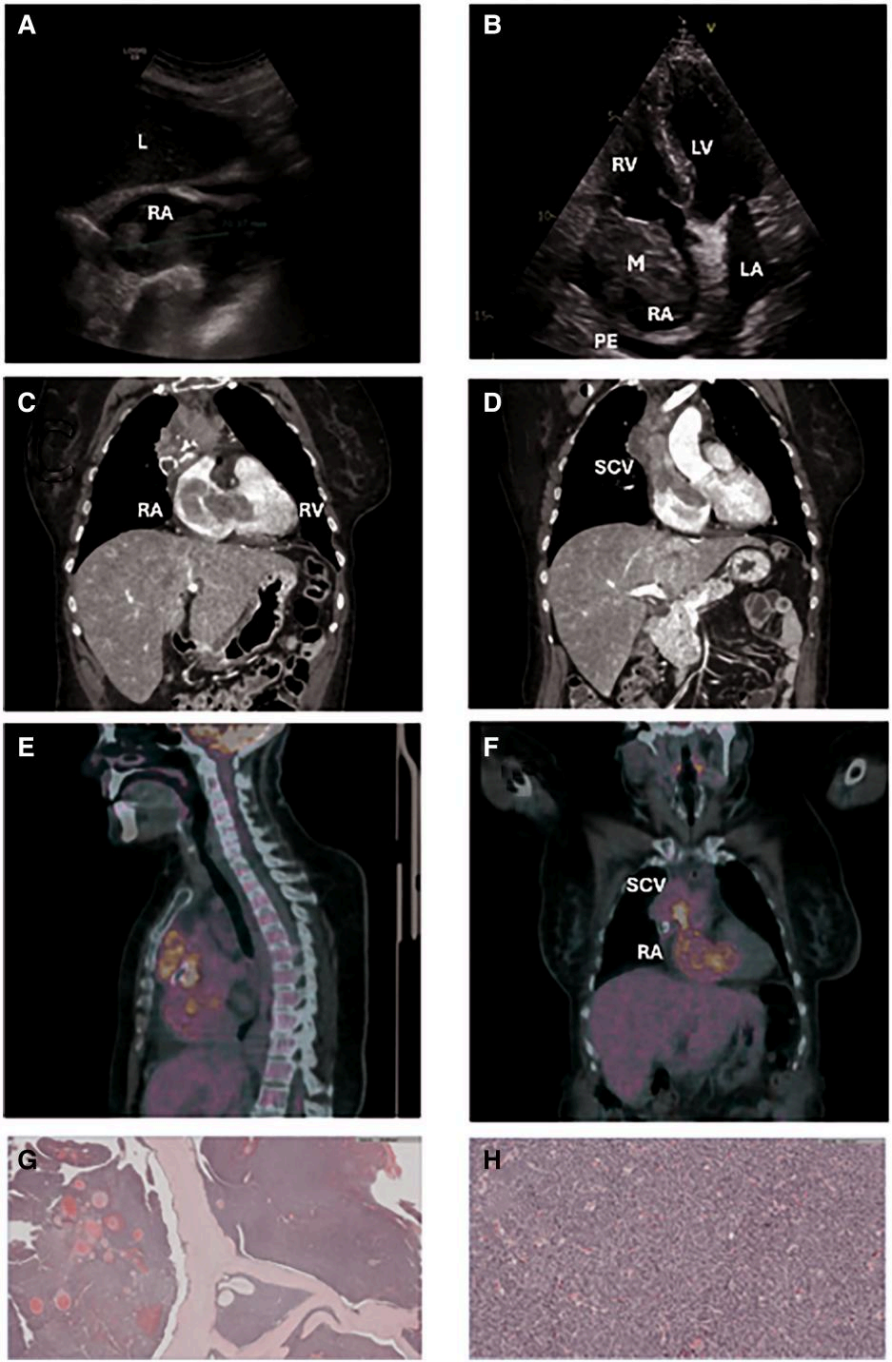
(*A*, *B*) Initially, an abdominal ultrasound (*A*, subcostal plane) was performed, revealing a 7.3 × 5.4 cm mass. Consequently, the on-call cardiologist performed a transthoracic echocardiogram (*B* and see Supplementary material online, *Video S1*, four-chamber plane), identifying a heterogeneous, poorly defined mass attached to the lateral wall of the right atrium, associated with a pericardial effusion, without being able to precisely delineate its extracardiac extension. (*C*, *D*) CT images were performed to evaluate the extracardiac extension of the mass. They reveal a heterogeneous, calcified mass originating from the anterior mediastinum, infiltrating the superior vena cava and the right atrium. (*E*, *F*) PET-CT images were requested to aid in the diagnostic orientation of the nature of the mass, revealing intense metabolic activity, consistent with its aggressive presentation and malignant behaviour. (*G*, *H*) Pathology of the mass. An intense lymphoplasmacytic infiltrate with areas of sclerosis is evident. However, the entire surgical specimen could not be analysed, making it impossible to rule out the coexistence of anatomopathological findings suggestive of malignancy, especially given the aggressive behaviour and intense metabolic uptake observed. M, mass; L, liver; LA, left atria; LV, left ventricle; PE, pericardial effusion; RA, right atria; RV, right ventricle; SCV, superior cava vein.

The patient underwent surgery involving partial resection of the mass, SCV excision, right atrial roof reconstruction, and anastomosis using a bovine xenograft to the left innominate vein, followed by adjuvant radiotherapy. Six months post-procedure, her clinical condition remained stable, and the abdominal symptoms improved, attributed to tricuspid stenosis causing congestive hepatomegaly.

Thymosarcomas, rare tumours accounting for <1% of thymic neoplasms, are often diagnosed at advanced stages due to their aggressive, invasive behaviour. Their clinical presentation may mimic thymomas, but they often exhibit necrosis, coarse calcifications, and poorly defined margins on imaging.^1^ Definitive diagnosis is challenging because the heterogeneity of thymic tumours complicates classification, and pathological analysis is often inconclusive when the entire surgical specimen cannot be evaluated. Therefore, a multimodal imaging approach, including computed tomography (CT), positron emission tomography (PET)-CT, and biopsy, is essential to accurately determine the tumour’s nature and origin.^2^

Treatment involves a multimodal approach. Surgical resection is the cornerstone, supplemented by adjuvant radiotherapy to manage residual disease and chemotherapy (e.g. platinum-based regimens with doxorubicin or cyclophosphamide) for advanced or inoperable cases.^2,3^ The prognosis remains poor, with survival outcomes primarily determined by patient age, tumour stage, and the potential for surgical resection.

## Supplementary Material

ytae699_Supplementary_Data

## Data Availability

No new data were created or analysed in this study.

## Notes

**Consent:** The authors confirm that written consent for submission and publication of this case report, including images and associated text, has been obtained from the patient in line with COPE guidance.

**Funding:** This research did not receive any specific grants.
